# Cognitive-affective configuration of university students’ continuance intention in online learning

**DOI:** 10.3389/fpsyg.2026.1816375

**Published:** 2026-06-02

**Authors:** Yizhen Wang, Xiaowei Chen

**Affiliations:** School of Arts, Zhejiang Shuren University, Hangzhou, China

**Keywords:** affective appraisal, BiLSTM-attention, cognitive-affective configuration, continuance intention, cross-sectional modelling, online learning

## Abstract

**Introduction:**

Most studies of continuance intention in online learning ask which psychological variables predict it. This study asks a different question: how are those variables arranged in relation to one another? Drawing on TAM, ECM, and a Control-Value-informed perspective, we treat continuance intention as a cognitive-affective configuration rather than as a belief-to-intention pathway dominated by a single variable.

**Methods:**

Survey data from 421 university students were analysed using five comparative models: BiLSTM, BiLSTM-Attention, CNN-BiLSTM, Transformer, and Random Forest.

**Results:**

The retained four-construct solution showed high internal consistency and strong convergent validity, although discriminant validity required cautious interpretation, particularly for the relationship between Affective Appraisal and Continuance Intention. Predictive performance was consistently strong across models (mean AUC = 0.936–0.954). Although the Friedman test detected overall variation in AUC, no pairwise comparison survived multiple-comparison correction. The BiLSTM-Attention model showed strong illustrative performance, but was interpreted as an exploratory computational tool rather than as evidence of temporal, recursive, or causal psychological structure.

**Discussion:**

The findings suggest that continuance intention in online learning is associated with a closely connected pattern of usefulness, trust, and affective appraisal rather than with usefulness alone. Practically, the results imply that platform design should extend beyond functional optimisation to include trust-building and support for positive evaluative experience.

## 1 Introduction

Students in online learning environments face a specific motivational problem that classroom learners do not: they must decide, often without much external prompting, whether continued participation is worth it. That decision involves both cognitive assessment—is this platform useful, is it reliable?—and affective evaluation—does using it feel satisfying, enjoyable? Most predictive studies of continuance intention treat these as competing variables in a regression. This study takes a different starting point: the question is not which variable wins, but how they are arranged in relation to one another.

Within educational psychology, Control-Value Theory (CVT) offers a relevant framework. CVT proposes that achievement emotions—enjoyment, anxiety, pride, boredom—arise from learners’ appraisals of perceived control and subjective value, and that these emotions in turn shape motivation, self-regulation, and persistence (Pekrun, 2006, 2024; Pekrun and Perry, 2014). Evidence from large-scale surveys during the COVID-19 pandemic confirms that emotional appraisals are systematically associated with engagement and outcomes in technology-mediated contexts (Raccanello et al., 2022). Research on technology adoption has addressed overlapping territory through TAM and ECM, which emphasise perceived usefulness, satisfaction, trust, and post-use confirmation (Davis, 1989; Bhattacherjee, 2001; Roca et al., 2006). These frameworks have a good empirical track record, but they are typically tested through additive regression models—models that can tell you whether each construct predicts continuance, but not how the constructs are positioned relative to one another in that prediction. Recent theoretical work has pushed back against treating cognition and affect as independent predictors (Pekrun, 2024; Harley et al., 2019). Positive activating emotions can support self-directed learning; negative emotions may either interfere with or motivate regulatory effort, depending on how the learner appraises the situation (Artino and Jones, 2012). This suggests that the relevant question is not just “do affect and cognition both predict continuance?” but “which matters more, under what conditions, and how do they relate?” Conventional machine learning models answer the first question well. A BiLSTM-Attention architecture may offer an additional way to discuss model behaviour in a theory-informed manner, but in the present study the attention mechanism is treated only as part of the architecture rather than as the basis for a standalone analysis of construct salience.

The present study uses a BiLSTM-Attention model as an exploratory computational tool to examine cross-sectional predictions of continuance intention from perceived usefulness, perceived trust, and affective appraisal. The data are cross-sectional and no temporal or causal claims are made. The contribution is interpretive rather than confirmatory: the attention mechanism is treated as part of the model architecture, not as the basis for a standalone analysis of psychological hierarchy. Accordingly, the study does not use attention outputs as direct evidence of theoretical ordering or predictive superiority over simpler comparison models.

## 2 Literature review

### 2.1 Learner engagement from control-value and technology acceptance perspectives

Control-Value Theory (CVT) proposes that achievement emotions arise from learners’ appraisals of perceived control and subjective value, and that these emotions feed back into motivation, self-regulation, and persistence (Pekrun, 2006, 2024). This is not a minor theoretical point: if emotions influence the very appraisals that produce them, then modelling them as independent additive predictors may be solving a simpler problem than CVT describes. The question is here not to test CVT in general, but to consider how aspects of its structuremay be represented in empirical work. Research on technology adoption has tackled overlapping territory through TAM and ECM, which emphasise perceived usefulness, post-use confirmation, and satisfaction as drivers of continued use (Davis, 1989; Bhattacherjee, 2001; Roca et al., 2006). These models work well as predictive tools. What these regression-based approaches are less suited to show is how the constructs relate to one another within an overall evaluative pattern rather than as independent predictors. CVT provides a relevant interpretive framework here because it highlights the close interdependence among appraisals, achievement emotions, and persistence-related outcomes. TAM, by contrast, has traditionally foregrounded perceived usefulness in continuance research. Whether these perspectives can be read as complementary, competing, or differently emphasised is not easy to examine through regression coefficients alone. The empirical question addressed in the present study is therefore not whether a model confirms a theoretically predicted hierarchy, but whether cross-sectional model behaviour can be described and interpreted in relation to these frameworks.

### 2.2 BiLSTM-attention as an exploratory computational tool

BiLSTM networks process inputs bidirectionally, so that each position in a sequence is informed by both preceding and subsequent context (Alzubi, 2022). In the present study, BiLSTM is used not to model temporal change, but to examine cross-sectional prediction under an architecture that can represent interdependence among inputs. An attention mechanism assigns differential emphasis within model training and may offer limited descriptive insight into model-internal processing (Ma et al., 2025; Ong et al., 2024). However, such weights are model artefacts rather than direct measures of psychological importance, and no causal inference is implied. Accordingly, the BiLSTM-Attention model is included as an exploratory architecture whose behaviour may be discussed cautiously in relation to theory, rather than as a basis for formal claims about construct ordering or psychological structure.

### 2.3 Gaps in cross-sectional modelling of cognitive-affective organisation

Computational approaches to educational data have advanced rapidly, and hybrid and ensemble models have shown strong predictive performance across learning analytics tasks (Farhana et al., 2022; Kusumawardani and Syukron Abu Ishaq Alfarozi, 2023). What is less common is work that connects the outputs of these models to established psychological frameworks in a way that makes the connection empirically interpretable. The TAM and ECM traditions have been tested mainly through regression-based models, which can establish whether each construct predicts an outcome but cannot show how constructs relate to one another structurally. By contrast, CVT highlights the close interdependence between control- and value-related appraisals, achievement emotions, and persistence-related outcomes. Whether attention-based architectures can support cautious, theory-informed discussion of model behaviour in this context has not been examined in the technology acceptance literature. That is the specific gap this study addresses. The present study uses a BiLSTM-Attention model as an exploratory tool to examine cross-sectional prediction of continuance intention from perceived usefulness, trust, and affective appraisal, and to discuss the resulting model behaviour cautiously in relation to CVT.

## 3 Methods

### 3.1 Research framework and variables

This study examined university students’ continuance intention in online learning using a cross-sectional modelling approach. Variables were organised using constructs from TAM (Davis, 1989) and ECM (Bhattacherjee, 2001) reinterpreted through a Control-Value theoretical lens. No temporal, recursive, or causal claims are made.

Four construct domains were specified. Perceived usefulness was treated as a value-related cognitive evaluation of platform utility. Perceived trust was treated as an evaluation of platform reliability and credibility. Satisfaction- and enjoyment-related items were modelled jointly as a single Affective Appraisal domain, consistent with CVT’s treatment of positive achievement emotions as a unified evaluative response to learning outcomes. As reported in the Results section, the four items showed strong internal consistency (*α* = 0.97) and adequate convergent validity (AVE = 0.88), supporting this specification. Continuance intention was defined as self-reported willingness to continue using the platform.

All constructs were measured using items adapted from validated instruments (Supplementary Table S1) on a five-point Likert scale (1 = strongly disagree; 5 = strongly agree). The original questionnaire included 28 candidate items. Items were retained on the basis of conceptual relevance, redundancy reduction, and psychometric adequacy for the present analytical framework. This process yielded 11 final items across four constructs: Perceived Usefulness (3), Perceived Trust (2), Affective Appraisal (4), and Continuance Intention (2). Internal consistency (Cronbach’s *α*) and discriminant validity (HTMT) are reported in the Results section, along with CFA loadings, fit indices, composite reliability, and average variance extracted.

### 3.2 Participants and data collection

Participants were undergraduate students in blended, theory-oriented courses at a comprehensive university in eastern China. The final sample was 421 students from education, management, and information technology programmes, providing disciplinary variation in background and digital learning experience. Data were collected via Wenjuanxing between March and May 2023 using stratified sampling. Of 500 distributed questionnaires, 421 valid responses were retained after screening for uniform response patterns, completion times under 120 s, and substantial missing data, yielding an effective response rate of 84.2%. The sample was 57.9% female and 42.1% male (M_age = 20.7 years, SD = 1.4).

### 3.3 Data processing and analytical procedure

The analytical procedure followed a structured and reproducible cross-sectional pipeline, including feature definition, preprocessing, model training, and comparative evaluation.

#### 3.3.1 Feature construction and outcome definition

Nine item-level predictors were used as inputs: three perceived usefulness items, two perceived trust items, and four affective appraisal items (combining satisfaction and enjoyment). The two continuance intention items were used only to define the target variable and were not included in the predictor set. Continuance intention was operationalised as the mean of the two CI items. Participants with a mean score of 4 or above (out of 5) were coded as intending to continue (1); those below 4 were coded as not intending to continue (0). This produced a moderately balanced target distribution (65.3% positive cases).

#### 3.3.2 Preprocessing and modelling strategy

Missing values were negligible and handled through listwise deletion. Input features were standardised (*z*-score) within each training fold and applied to the corresponding test fold. Five-fold stratified cross-validation was used: each fold held out approximately 20% of data for testing, and every observation served as test data once. The core model was a Bidirectional Long Short-Term Memory (BiLSTM) network with an attention mechanism. The bidirectional structure allows each input position to draw on both preceding and subsequent context. The BiLSTM-Attention model is used here as an exploratory architecture for cross-sectional prediction rather than as a basis for formal claims about feature hierarchy, temporal priority, or psychological mechanism. Five models were compared under identical data and validation procedures: BiLSTM-Attention, standard BiLSTM, CNN-BiLSTM, Transformer, and Random Forest. All models used the same nine predictors and binary target.

#### 3.3.3 Model training and evaluation

Neural models were trained using Adam optimisation (learning rate = 0.001) with binary cross-entropy loss. Early stopping was applied after 10 epochs without improvement, with a maximum of 50 epochs and batch size of 64. Performance was evaluated using accuracy, precision, recall, F1-score, and AUC. Mean performance across folds was reported. Friedman tests were used for omnibus comparisons; pairwise differences were tested with Wilcoxon signed-rank tests and corrected for multiple comparisons where appropriate. Although the BiLSTM-Attention architecture includes an attention mechanism, the present study did not conduct a standalone inferential analysis of attention weights. Accordingly, the attention component is interpreted only as part of the model architecture and is not treated as evidence of temporal priority, psychological centrality, or causal mechanism.

### 3.4 Ethical considerations

The study was conducted in accordance with the Declaration of Helsinki and institutional guidelines for human participants’ research. Ethical approval was granted by the Institutional Ethics Committee of the Zhejiang Institute of Communications [Approval No. REC/02/2018 (RZ-2018-07)]. Oral informed consent was obtained prior to data collection. Participants were informed of the study purpose, the voluntary nature of participation, confidentiality measures, and their right to withdraw without penalty. Written consent was not required given the minimal risk involved. Anonymised data and analysis scripts are available on reasonable academic request.

## 4 Results

### 4.1 Measurement results of the four-construct solution

All four constructs showed strong internal consistency (Cronbach’s *α* = 0.90–0.97) and satisfactory convergent validity (CR = 0.90–0.97; AVE = 0.82–0.91). Standardised CFA loadings ranged from 0.87 to 0.96; PT showed the lowest item loadings (0.87–0.94) among the four constructs, consistent with its two-item structure. Discriminant validity presented a mixed picture (Table 1, Panel A). As shown in Table 2, Panel A, HTMT values ranged from 0.75 (PU-PT) to 0.93 (Affect-CI). Two pairs exceeded the 0.85 threshold: Affect-CI at 0.93 and PT-Affect at 0.91. Critically, bootstrap lower bounds confirmed these exceedances even under sampling uncertainty (Affect-CI 95% CI: 0.90–0.95; PT-Affect 95% CI: 0.88–0.94) (Table 2, Panel B), indicating that the proximity between affective and cognitive-appraisal constructs is not attributable to estimation error. The remaining four pairs fell below 0.85, with PU-PT showing the clearest separation (HTMT = 0.75, CI: 0.67–0.82). PT-CI (HTMT = 0.87) occupied an intermediate position.

**Table 1:** Reliability, convergent validity, and CFA results for the four-construct solution.

Panel A. Construct-level reliability and convergent validity.					
Construct	n items	Cronbach’s *α*	CR	AVE	Standardised loading range
PU	3	0.9441	0.9449	0.8513	0.8809–0.9526
PT	2	0.8987	0.9016	0.8211	0.8736–0.9376
Affect	4	0.9677	0.9678	0.8827	0.9361–0.9459
CI	2	0.9547	0.9548	0.9135	0.9495–0.9619

Panel B. Global CFA fit indices.	
Fit index	Value
*χ* ^2^	266.2946
df	38
*χ*^2^/df	7.0078
CFI	0.9806
TLI	0.9720
RMSEA	0.0878
GFI	0.9775
AGFI	0.9675
NFI	0.9775

**Table 2:** Discriminant validity of the four-construct solution.

Panel A. HTMT matrix.				
Construct	PU	PT	Affect	CI
PU	1.0000	0.7500	0.7997	0.7861
PT	0.7500	1.0000	0.9119	0.8683
Affect	0.7997	0.9119	1.0000	0.9290
CI	0.7861	0.8683	0.9290	1.0000

Panel B. Bootstrap 95% confidence intervals for HTMT values				
Construct A	Construct B	95% CI lower	95% CI upper	Bootstrap mean
Affect	CI	0.9036	0.9508	0.9287
PT	Affect	0.8794	0.9408	0.9114
PT	CI	0.8273	0.9021	0.8676
PU	Affect	0.7313	0.8650	0.8004
PU	CI	0.7150	0.8503	0.7861
PU	PT	0.6736	0.8229	0.7506

As shown in Table 1, Panel B, CFA fit was strong on relative indices (CFI = 0.980, TLI = 0.972, NFI = 0.978, GFI = 0.978, AGFI = 0.968) but showed modest strain on absolute indices (*χ*^2^/df = 7.01, RMSEA = 0.088). The latter reflects the well-known sensitivity of *χ*^2^ to sample size in larger surveys; the CFI/TLI values above 0.97 indicate acceptable fit by most applied standards (Hair et al., 2010).

Taken together, these results support the use of the four-construct solution as a workable analytical framework, while also indicating that the boundaries among Affect, trust, and continuance intention should be interpreted cautiously.

### 4.2 Comparative predictive performance across models

Five models were compared under five-fold stratified cross-validation: BiLSTM, BiLSTM-Attention, CNN-BiLSTM, Transformer, and Random Forest. As shown in Table 3, mean ± SD values are reported for accuracy, precision, recall, F1-score, and AUC across folds. All models performed well (AUC range: 0.936–0.954; accuracy range: 0.879–0.891). Transformer achieved the highest AUC (0.954) and recall (0.928); CNN-BiLSTM showed the highest accuracy (0.891) and F1-score (0.917); BiLSTM-Attention led in precision (0.917). Random Forest, included as a non-deep-learning comparison model, scored lowest across all five metrics—most notably in AUC (0.936) and precision (0.898), with the highest standard deviation in precision (SD = 0.041), indicating less stable predictions across folds. The two deep learning variants and Transformer consistently showed higher mean values than this baseline on most indicators.

**Table 3:** Predictive performance of five models under five-fold stratified cross-validation.

Model	Accuracy	Precision	Recall	F1-score	AUC
	(mean ± SD)				
BiLSTM	0.8875 ± 0.0169	0.9128 ± 0.0211	0.9159 ± 0.0254	0.9140 ± 0.0132	0.9466 ± 0.0146
BiLSTM-Attention	0.8901 ± 0.0224	0.9165 ± 0.0245	0.9159 ± 0.0314	0.9158 ± 0.0176	0.9473 ± 0.0152
CNN-BiLSTM	0.8913 ± 0.0125	0.9134 ± 0.0184	0.9218 ± 0.0284	0.9171 ± 0.0104	0.9476 ± 0.0132
Random Forest	0.8785 ± 0.0184	0.8979 ± 0.0408	0.9218 ± 0.0338	0.9085 ± 0.0118	0.9356 ± 0.0192
Transformer	0.8862 ± 0.0193	0.9028 ± 0.0334	0.9276 ± 0.0358	0.9141 ± 0.0145	0.9541 ± 0.0153

A Friedman test detected overall variation in AUC across models (*χ*^2^ = 16.44, *p* = 0.003). However, as shown in Table 4, no pairwise comparison survived correction for multiple testing (Bonferroni, Holm, or FDR-BH). The closest pair was BiLSTM versus Random Forest (Wilcoxon *W* = 0.0, raw *p* = 0.0625; FDR-adjusted *p* = 0.089)—suggesting a trend but not confirming a stable difference within the present sample. All other comparisons showed *p*-values well above significance thresholds. BiLSTM-Attention was not selected because it outperformed others on AUC. It was retained as an exploratory architecture that allowed a theory-informed discussion of model behaviour under the same validation framework, rather than as evidence of clear architectural superiority.

**Table 4:** Post-hoc Wilcoxon tests for AUC comparisons with multiple-comparison correction (*p*).

Comparison	Statistic	*p*				Significant after correction
		Raw	Bonferroni	Holm	FDR-BH	
BiLSTM vs. BiLSTM-Attention	1.0	0.2500	1.0000	0.7500	0.3125	No
BiLSTM vs. CNN-BiLSTM	6.0	0.7500	1.0000	1.0000	0.8333	No
BiLSTM vs. Random Forest	0.0	0.0625	0.6250	0.6250	0.0893	No
BiLSTM vs. Transformer	0.0	0.0625	0.6250	0.6250	0.0893	No
BiLSTM-Attention vs. CNN-BiLSTM	7.0	1.0000	1.0000	1.0000	1.0000	No
BiLSTM-Attention vs. Random Forest	0.0	0.0625	0.6250	0.6250	0.0893	No
BiLSTM-Attention vs. Transformer	0.0	0.0625	0.6250	0.6250	0.0893	No
CNN-BiLSTM vs. Random Forest	0.0	0.0625	0.6250	0.6250	0.0893	No
CNN-BiLSTM vs. Transformer	0.0	0.0625	0.6250	0.6250	0.0893	No
Random Forest vs. Transformer	0.0	0.0625	0.6250	0.6250	0.0893	No

### 4.3 Illustrative performance of the BiLSTM-attention model

**Figure 1:**
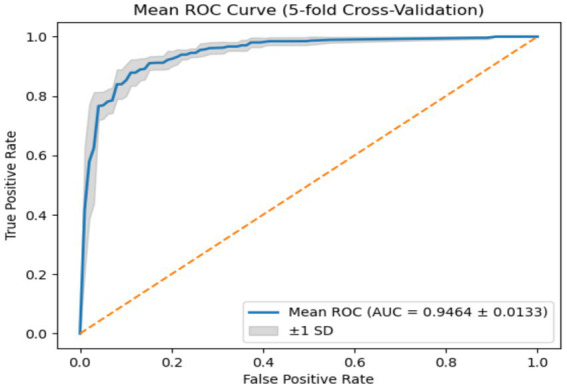
Mean ROC curve of the BiLSTM-Attention model across five-fold stratified cross-validation. The model achieved a mean AUC of 0.9464 ± 0.0133.

**Figure 2:**
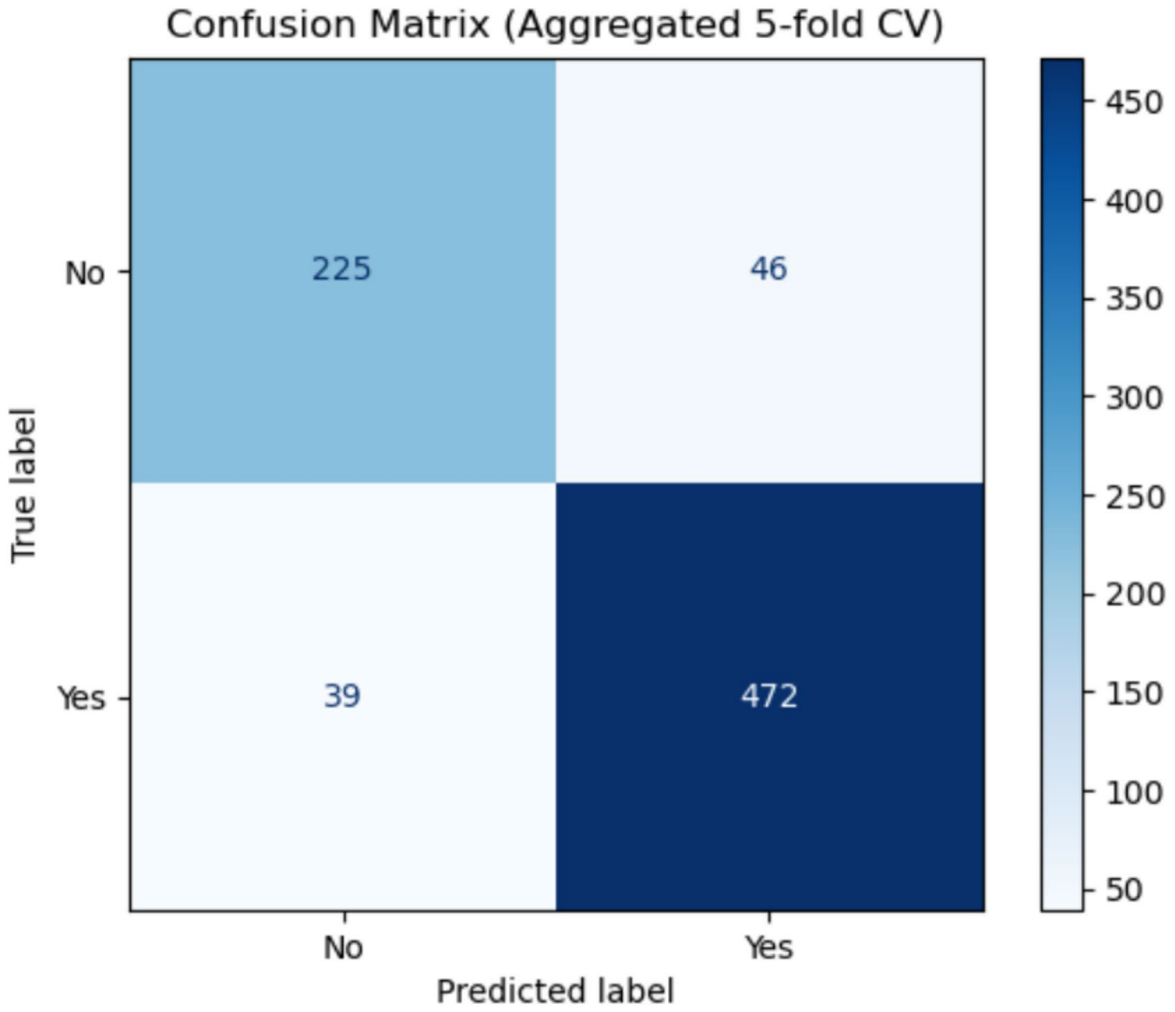
Aggregated confusion matrix of the BiLSTM-Attention model across five-fold stratified cross-validation.

Aggregated predictions across folds showed strong discriminative performance. Figure 1 shows the mean ROC curve, which yielded an AUC of 0.9464 ± 0.0133. The relatively narrow ±1 SD band suggests stable performance across folds. As shown in Figure 2, the aggregated confusion matrix indicated a class distribution of 511 positive and 271 negative cases in the ground truth (ratio ≈ 1.9:1). The model correctly classified 472 of 511 positive cases (sensitivity = 92.4%) and 225 of 271 negative cases (specificity = 83.0%). False positives (*n* = 46) slightly exceeded false negatives (*n* = 39), yielding a positive predictive value of 91.1% and a negative predictive value of 85.2%. Overall, this pattern suggests a modest tendency to classify cases as high continuance intention, which may partly reflect the class distribution in the present sample.

## 5 Discussion

### 5.1 Summary of the main findings

Three findings warrant discussion. First, discriminant validity showed a graded but mixed pattern. Affective Appraisal and Continuance Intention (HTMT = 0.93), and Perceived Trust and Affective Appraisal (HTMT = 0.91), both exceeded the 0.85 threshold with bootstrap lower bounds well above it; Perceived Trust and Continuance Intention occupied an intermediate position (HTMT = 0.87); PU-PT showed the clearest separation (HTMT = 0.75). This pattern suggests that affective appraisal, trust, and continuance intention were empirically close in the present dataset, and that their boundaries should therefore be interpreted cautiously. Second, all five models performed comparably (AUC range: 0.936–0.954). Transformer led on AUC and recall; CNN-BiLSTM led on accuracy and F1-score; Random Forest showed the lowest mean values across the five metrics. However, no pairwise comparison survived multiple-comparison correction, indicating that no single model demonstrated stable superiority in the present sample. Third, the BiLSTM-Attention model was retained as an illustrative model for examining predictive behaviour in greater detail. Its strong classification performance is discussed below, while any model-internal behaviour associated with the BiLSTM-Attention architecture is interpreted cautiously as exploratory rather than confirmatory.

### 5.2 Comparative analysis with existing literature

The findings suggest that continuance intention in online learning reflects a cognitive-affective evaluative pattern rather than a cognition-first outcome. This aligns with CVT, which holds that appraisals of control and value are related to achievement emotions and engagement outcomes (Pekrun, 2006, 2024; Artino and Jones, 2012). Perceived usefulness remained relevant, but affective evaluation and trust were closely tied to continuance intention—suggesting that persistence-related judgment involves broader evaluative organisation than usefulness alone.

This pattern also fits with TAM and ECM, both of which foreground usefulness, trust, confirmation, and post-use evaluation (Bhattacherjee, 2001; Davis, 1989; Roca et al., 2006). The high proximity between Affect and CI, and between Affect and trust, indicates that affective and intentional judgments are empirically intertwined in self-report data. The study thus extends prior work by suggesting that these factors are less sharply differentiated than linear acceptance models imply.

The results do not support claims about temporal process, recursive appraisal, or causal ordering. They do support a cross-sectional account: continuance intention is associated with a configuration of value-related, trust-related, and affective evaluations. This reading matters because discriminant validity was mixed—the four-construct solution is analytically useful but not fully resolved.

Methodologically, all five models performed strongly, yet no pairwise superiority survived multiple-comparison correction. The study should not be read as showing that one architecture outperformed the others. Its contribution is a theory-informed comparative framework for examining cognitive-affective organisation in cross-sectional data, consistent with recent work emphasising transparency and theoretical alignment in educational machine learning (Akgül and Uymaz, 2022; Hu et al., 2024; Farhana et al., 2022; Kusumawardani and Syukron Abu Ishaq Alfarozi, 2023). The attention mechanism is interpreted cautiously as a model-internal component of the BiLSTM-Attention architecture rather than as an independent source of psychological evidence (Jain and Wallace, 2019; Serrano and Smith, 2019; Wiegreffe and Pinter, 2019). No standalone ordering of constructs is inferred from attention outputs, and no claim is made that the model confirms psychological structure.

### 5.3 Theoretical and practical implications

Theoretically, this study reframes continuance intention as a cognitive-affective configuration rather than a usefulness-driven outcome alone. Within cross-sectional self-report data, this does not overturn TAM or ECM; rather, it clarifies how usefulness, trust, and affective appraisal are jointly implicated in students’ willingness to persist (Bhattacherjee, 2001; Davis, 1989). The discriminant validity results showed a graded HTMT pattern (Affect-CI: 0.93; PT-Affect: 0.91; PT-CI: 0.87; PU-PT: 0.75), which is broadly consistent with the view that continuance intention in online learning involves closely connected cognitive and affective evaluations rather than sharply separated domains. Usefulness remains relevant, while trust and affective appraisal also appear closely implicated in this judgment.

Methodologically, the absence of stable pairwise superiority after multiple-comparison correction (Tables 3, 4) means no single architecture can be declared dominant. This constraint shapes the study’s contribution: rather than a performance contest among models, the work offers a theory-informed comparative framework for examining cognitive-affective organisation in cross-sectional data. The BiLSTM-Attention model’s robust classification performance (Figures 1, 2) should be read in this spirit. Its attention mechanism may reflect model-internal weighting during optimisation, but in the present study it was not analysed as a standalone inferential result and therefore should not be treated as evidence of causal priority or validated psychological hierarchy (Ma et al., 2025; Ong et al., 2024; Bodria et al., 2023).

Practically, sustaining online learning engagement may require more than functional optimisation. Interventions targeting perceived trust, expectation alignment, and positive evaluative experiences deserve comparable attention. Learners persist not only because a platform is useful, but because it feels reliable and affectively supportive—a consideration that points toward more human-centred design. The model’s asymmetric performance—higher accuracy in identifying high-continuance than low-continuance cases—also suggests that future early-warning systems for at-risk learners may benefit from incorporating behavioural trace data rather than relying on self-report measures alone.

### 5.4 Limitations and future research

Three limitations warrant attention. First, the cross-sectional, self-report design limits causal inference and generalisability to the sampled context. Second, the empirical overlap among Affective Appraisal, Perceived Trust, and Continuance Intention (Tables 1, 2) indicates that construct boundaries are not fully resolved; the four-construct solution is analytically useful but should not be treated as a confirmed latent structure. Third, the absence of pairwise model superiority after correction restricts claims about algorithmic dominance.

Future work could address these constraints in several directions. Longitudinal or experience-sampling designs would allow examination of how cognitive-affective evaluations unfold over time. Integrating behavioural trace data could reduce dependence on self-report overlap and provide more direct engagement indicators. Expanding the item pool for affective constructs may sharpen differentiation between related forms of positive evaluation. Cross-context comparison—whether similar patterns emerge across platforms, age groups, or disciplines—would also strengthen external validity.

## 6 Conclusion

This study examined continuance intention in online learning as a cognitive-affective configuration, using a theory-informed comparative modelling framework. The four-construct solution provided a workable basis for analysis, although some construct boundaries remained empirically close and discriminant validity therefore required cautious interpretation. Across five models, predictive performance was consistently strong, yet no pairwise superiority remained significant after correction. The BiLSTM-Attention model showed robust classification behaviour, with its attention mechanism best understood as a descriptive, model-internal supplement rather than evidence of causal or temporal structure. Continuance intention in online learning is not usefulness alone. The findings indicate that it involves a broader evaluative pattern in which usefulness, trust, and affective appraisal all play a role. Rather than advancing strong structural or algorithmic claims, this study offers a theory-informed account of how these factors relate to students’ willingness to persist.

## Data Availability

The raw data supporting the conclusions of this article will be made available by the authors, without undue reservation.

## References

[ref1] Akgül Y. Uymaz A. O. (2022). Facebook/Meta usage in higher education: a deep learning-based dual-stage SEM-ANN analysis. Educ. Inf. Technol. 27, 9821–9855. doi: 10.1007/s10639-022-11012-9, 35399779 PMC8979783

[ref2] Alzubi J. A. (2022). Student academic performance prediction using LSTM and GRU: a comparative study. Educ. Inf. Technol. 27, 2175–2191. doi: 10.1007/s10639-021-10727-x

[ref3] Artino A. R. Jones K. D. (2012). Exploring the complex relations between achievement emotions and self-regulated learning behaviors in online learning. Internet High. Educ. 15, 170–175. doi: 10.1016/j.iheduc.2012.01.006

[ref4] Bhattacherjee A. (2001). Understanding information systems continuance: an expectation-confirmation model. MIS Q. 25, 351–370. doi: 10.2307/3250921

[ref5] Bodria F. Giannotti F. Guidotti R. Naretto F. Pedreschi D. Rinzivillo S. (2023). Benchmarking and survey of explanation methods for black box models: F. Bodria et al. Data Min. Knowl. Discov. 37, 1719–1778. doi: 10.1007/s10618-023-00933-9

[ref6] Davis F. D. (1989). Perceived usefulness, perceived ease of use, and user acceptance of information technology. MIS Q. 13, 319–340. doi: 10.2307/249008

[ref7] Farhana M. Haque M. A. Karim R. (2022). Student performance prediction model using machine learning algorithms. Educ. Inf. Technol. 27, 3931–3950. doi: 10.1007/s10639-021-10707-3

[ref8] Harley J. M. Pekrun R. Taxer J. L. Gross J. J. (2019). Emotion regulation in achievement situations: an integrated model. Educ. Psychol. 54, 106–126. doi: 10.1080/00461520.2019.1587297

[ref8001] Hair J. F. Black W. C. Babin B. J. Anderson R. E. (2010). Multivariate data analysis: A global perspective. Upper Saddle River, NJ: Pearson Prentice Hall.

[ref9] Hu X. Goh Y. M. Tay J. (2024). Construction professionals’ perspectives of adaptive learning adoption: an SEM-machine learning approach. Eng. Constr. Archit. Manag. 33, 1452–1480. doi: 10.1108/ECAM-07-2024-0896, 35579975

[ref10] Jain S. Wallace B. C. (2019). Attention is not explanation. In. Burstein J. Doran C. Solorio T. (Eds.), Proceedings of the 2019 Conference of the North American Chapter of the Association for Computational Linguistics: Human Language Technologies, vol. 1 (Long and Short Papers)(3543–3556). Minneapolis, Minnesota, USA: Association for Computational Linguistics. doi: 10.18653/v1/N19-1357

[ref11] Kusumawardani R. Syukron Abu Ishaq Alfarozi M. (2023). Deep learning models for knowledge tracing: a comparative analysis. Educ. Inf. Technol. 28, 2051–2070. doi: 10.1007/s10639-022-11133-5

[ref12] Ma J. Wang P. Li B. Wang T. Pang X. S. Wang D. (2025). Exploring user adoption of ChatGPT: a technology acceptance model perspective. Int. J. Hum. Comput. Interact. 41, 1431–1445. doi: 10.1080/10447318.2024.2314358

[ref14] Ong A. K. S. Prasetyo Y. T. Tapiceria R. P. K. M. Nadlifatin R. Gumasing M. J. J. (2024). Factors affecting the intention to use COVID-19 contact tracing application “StaySafe PH”: integrating protection motivation theory, UTAUT2, and system usability theory. PLoS One 19:e0306701. doi: 10.1371/journal.pone.0306701, 39088508 PMC11293755

[ref15] Pekrun R. (2006). The control-value theory of achievement emotions: assumptions, corollaries, and implications for educational research and practice. Educ. Psychol. Rev. 18, 315–341. doi: 10.1007/s10648-006-9029-9

[ref16] Pekrun R. (2024). Control-value theory: from achievement emotion to a general theory of human emotions. Educ. Psychol. Rev. 36:83. doi: 10.1007/s10648-024-09909-7

[ref18] Pekrun R. Perry R. P. (2014). “Control-value theory of achievement emotions,” in International Handbook of Emotions in Education, eds. Pekrun R. Linnenbrink-Garcia L. (New York, NY: Routledge), 120–141.

[ref19] Raccanello D. Balbontín-Alvarado R. da Silva Bezerra D. Burro R. Cheraghi M. Dobrowolska B. . (2022). Higher education students’ achievement emotions and their antecedents in e-learning amid COVID-19 pandemic: a multi-country survey. Learn. Instr. 80:101629. doi: 10.1016/j.learninstruc.2022.101629, 35578734 PMC9095445

[ref20] Roca J. C. Chiu C. M. Martínez F. J. (2006). Understanding e-learning continuance intention: an extension of the technology acceptance model. Int. J. Hum.-Comput. Stud. 64, 683–696. doi: 10.1016/j.ijhcs.2006.01.003

[ref21] Serrano S. Smith N. A. (2019). “Is attention interpretable?” in Proceedings of the 57th Annual Meeting of the Association for Computational Linguistics (pp. 2931–2951). (Florence, Italy: Association for Computational Linguistics), doi: 10.18653/v1/P19-1282

[ref25] Wiegreffe S. Pinter Y. (2019). “Attention is not explanation,” in Proceedings of the 2019 Conference on Empirical Methods in Natural Language Processing and the 9th International Joint Conference on Natural Language Processing (EMNLP-IJCNLP), (pp. 11–20). Hong Kong, China: Association for Computational Linguistics. doi: 10.18653/v1/D19-1002

