# Supplementary material for: Cognitive-affective configuration of university students’ continuance intention in online learning
Source: Front Psychol. 2026 Jun 2;17:1816375. doi: 10.3389/fpsyg.2026.1816375 (PMC13271016; doi:10.3389/fpsyg.2026.1816375)
Supplement: Supplementary file 1 [file Table_1.docx]

**Supplementary Appendix**

Table S1. Final retained questionnaire items for the four-construct solution

| **Construct** | **Item Code** | **Questionnaire Item** | **Source** |
| --- | --- | --- | --- |
| **Perceived Usefulness** | PU1 | Using the smart learning platform can improve my learning efficiency. | Bhattacherjee (2001) |
|  | PU2 | Using the platform can improve the quality of my learning. |  |
|  | PU3 | I can find practical knowledge and information related to theory. |  |
| **Perceived Trust** | PT1 | The platform will not leak my private information. | Tan (2001) |
|  | PT2 | The learning materials provided are authoritative and reliable. |  |
| **Affective Appraisal** | AFF1 | I am satisfied with the learning experience on the platform. | Bhattacherjee (2001), Oliver (1980) |
|  | AFF2 | Using the platform makes me feel more relaxed, learn efficiently, and be happy. | Davis (1989), Tsang (2004) |
|  | AFF3 | Using the platform is an enjoyable and exciting process. |  |
|  | AFF4 | I am satisfied with the platform overall. |  |
| **Continuance Intention** | CI1 | If possible, I will frequently use the platform. | Bhattacherjee (2001) |
|  | CI2 | I will recommend the platform to others. |  |
